# Global gene expression changes in human embryonic lung fibroblasts induced by organic extracts from respirable air particles

**DOI:** 10.1186/1743-8977-9-1

**Published:** 2012-01-12

**Authors:** Helena Líbalová, Kateřina Uhlířová, Jiří Kléma, Miroslav Machala, Radim J Šrám, Miroslav Ciganek, Jan Topinka

**Affiliations:** 1 Department of Genetic Ecotoxicology, Institute of Experimental Medicine, Academy of Sciences of the Czech Republic, 142 20 Prague 4, Czech Republic; 2 Department of Biochemistry, Faculty of Science, Charles University, Albertov 2030, 128 40 Prague 2, Czech Republic; 3 Czech Technical University in Prague, Prague 2, Czech Republic; 4 Veterinary Research Institute, Brno, Czech Republic

**Keywords:** air pollution, complex mixtures, HEL cells, CYP1B1, AhR, gene expression profile

## Abstract

**Background:**

Recently, we used cell-free assays to demonstrate the toxic effects of complex mixtures of organic extracts from urban air particles (PM2.5) collected in four localities of the Czech Republic (Ostrava-Bartovice, Ostrava-Poruba, Karvina and Trebon) which differed in the extent and sources of air pollution. To obtain further insight into the biological mechanisms of action of the extractable organic matter (EOM) from ambient air particles, human embryonic lung fibroblasts (HEL12469) were treated with the same four EOMs to assess changes in the genome-wide expression profiles compared to DMSO treated controls.

**Method:**

For this purpose, HEL cells were incubated with subtoxic EOM concentrations of 10, 30, and 60 μg EOM/ml for 24 hours and global gene expression changes were analyzed using human whole genome microarrays (Illumina). The expression of selected genes was verified by quantitative real-time PCR.

**Results:**

Dose-dependent increases in the number of significantly deregulated transcripts as well as dose-response relationships in the levels of individual transcripts were observed. The transcriptomic data did not differ substantially between the localities, suggesting that the air pollution originating mainly from various sources may have similar biological effects. This was further confirmed by the analysis of deregulated pathways and by identification of the most contributing gene modulations. The number of significantly deregulated KEGG pathways, as identified by Goeman's global test, varied, depending on the locality, between 12 to 29. The Metabolism of xenobiotics by cytochrome P450 exhibited the strongest upregulation in all 4 localities and *CYP1B1 *had a major contribution to the upregulation of this pathway. Other important deregulated pathways in all 4 localities were ABC transporters (involved in the translocation of exogenous and endogenous metabolites across membranes and DNA repair), the Wnt and TGF-β signaling pathways (associated particularly with tumor promotion and progression), Steroid hormone biosynthesis (involved in the endocrine-disrupting activity of chemicals), and Glycerolipid metabolism (pathways involving the lipids with a glycerol backbone including lipid signaling molecules).

**Conclusion:**

The microarray data suggested a prominent role of activation of aryl hydrocarbon receptor-dependent gene expression.

## Background

Considerable efforts have been made to clarify the adverse effects of environmental pollution on human health [1]. Respirable ambient air particulate matter with an aerodynamic diameter < 2.5 μm (PM2.5) is a complex mixture consisting of a large number of chemicals, many of which are toxic and/or carcinogenic [2]. The mixtures of organic compounds to which the general population is exposed are not completely characterized since complex chemical analysis is very difficult. Investigations into the biological effects of ambient air particulate matter have involved a number of different approaches, including the study of particle-induced genotoxicity. Although hundreds of genotoxic compounds have been identified in ambient air, less than 25 of these compounds are routinely monitored [3]. Therefore, a biological approach based on specific toxic effects, such as direct or indirect reactivity with DNA or mutagenicity of complex mixture components might represent a suitable alternative [4,5]. The toxic effects of ambient air particulate matter (PM) are most frequently associated with chemicals bound onto the surface of the PM and/or with the particles themselves [6,7]. Some studies suggest that the genotoxic effects of PM are induced by polycyclic hydrocarbons (PAHs) and their derivatives forming the organic fraction of PM [1,8,9]. Other studies indicate that some metals forming PM may catalyze the oxidative damage of DNA [10-12]. Much less attention has been paid to nongenotoxic mechanisms of the toxic effects of chemicals bound onto PM2.5, although complex mixtures of air pollutants are known to contain various tumor promoters [13,14]. It has been repeatedly demonstrated that some PAHs, such as benzo[a]pyrene (BaP), form DNA adducts, after their metabolic activation by cytochrome P450 enzymes [15-18]. However, the PAHs, which activate aryl hydrocarbon receptor (AhR), induce several AhR-dependent nongenotoxic effects associated with tumor promotion [19,20]. PAHs have been reported to contribute to antiapoptotic effect of PM via activation of AhR in human bronchial epithelial cells [21] and AhR-dependent induction of cell proliferation, another hallmark of tumor promotion, after exposure to the extract of reference airborne particles has been described in liver epithelial cells [14]. Moreover, another group of PAHs (fluoranthene, pyrene) is known to exhibit tumor promoting activity via inhibition of intercellular communication [13,22].

Several attempts have been made to study the toxic effects of both artificial and real mixtures of environmental air pollutants, including PAHs, in various cell cultures [23]. The recent progress of "omics" technology in toxicology has allowed more insight into the mechanisms of the toxic effects of complex mixtures [24]. This technology offers the ability to query the entire genome after exposure to a complex mixture of compounds, permitting characterization of the biological effects of such exposure and the mechanisms of action involved. Significant attention has been paid to the global gene expression changes caused by complex mixtures, such as cigarette smoke and its condensate, diesel exhaust and carbon black. However, only a few studies have dealt with ambient dust particles (reviewed in [24]). The genome-wide study, dealing with particles from urban dust (standardized SRM1649a) in a human cell line in vitro, indicated deregulation of genes involved in DNA repair, peroxisome proliferation, metabolism and changes in tissue growth factors and oncogenes [25]. In human aortic endothelial cells exposed to the ambient particular matter, the modulation of gene expression included upregulation of metabolism of xenobiotics and proinflammatory responses [26].

In this study, the toxicogenomic approach was used to identify genes and particularly the biological pathways involved in the action of mixtures of organic air pollutants adsorbed onto respirable air particles (PM2.5). As a model system, human embryonic lung fibroblasts (HEL) which have been repeatedly shown to be a suitable model for toxicity studies of individual compounds as well as artificial and environmental mixtures, were used [9,27]. Importantly, embryonic fibroblasts have distinctive differentiation status compared with other lung cell models and therefore, unique gene expression changes might be expected. Changes in the whole genome expression profiles induced by extractable organic matter (EOM) from the PM2.5 particles in HEL cells were analyzed at subtoxic EOM concentrations and significantly deregulated genes and biological processes were identified. Moreover, changes in gene expression profiles for various localities (differing by the sources and extent of air pollution) were compared with the aim of identifying exclusive changes in gene transcription profiles corresponding to the air pollution exposure.

## Results

### Air sampling

The occurrence of organic compounds in the air is dependent on their physical properties, there are present in the gas phase, partially or completely adsorbed to the particles present in the air. This fact complicates the procedures for air sampling and the interpretation of the observed concentrations or toxic effects. PAHs with two to three cycles are present in the air under normal physical conditions in the gas phase (partly but can also be adsorbed on air particles, e.g. fluoranthene), PAHs with four cycles (e.g., pyrene) are distributed both in the gas and particulate phase, and PAHs with five or more cycles (benzo[*a*]pyrene, benzofluoranthenes, dibenzoanthracenes and dibenzopyrenes) are almost entirely adsorbed on particles [28-30]. This study was also focused on the determination of organic extractable compounds bound to the particulate matter in the air, which in terms of genotoxicity, carcinogenicity and dioxin-like toxicity represent the highest risk.

The basic characteristics of PM2.5 sampling such as GPS coordinates, volume of sampled air, concentrations of PM2.5 and EOM in all localities are summarized in Table 1. The highest air pollution level in terms of PM2.5 was found in the industrial area of Ostrava-Bartovice (1.5-fold and more than 3-fold higher than in Ostrava-Poruba and Trebon, respectively).

**Table 1:** Basic characteristics of PM2.5 sampling in various localities of the Czech Republic

Locality [GPS coordinates]	Sampling period	Air volume [m^3^]	PM [μg/m^3^]	EOM **[μg/m^3^**]
Ostrava-Bartovice	1.3.-4.4. 09	29,900	36.7	13.0
[49°48'07"N, 18°20'56"E]				
Ostrava-Poruba	1.3.-31.3. 09	35,200	25.8	8.05
[49°48'07"N, 18°20'56"E]				
Karvina	1.4.-5.5. 09	47,400	n.a.*	9.16
[49°48'07"N, 18°20'56"E]				
Trebon	19.11.-17.12. 08	44,700	11.4	4.15
[49°00'15"N, 14°45'56"E]				

*Missing for technical reasons

### Chemical characterization of ambient air particulate matter

To evaluate the chemical characterization of ambient air particulate matter (PM_2.5_), many classes of organic contaminants were analyzed (Additional file 1). The highest concentrations were found for n-alkanes (77.4 - 89.5 ng/m^3^), ten U.S. EPA PAHs (parent PAHs prioritized by U.S. EPA, ranged from 5.89 to 76.3 ng/m^3^), other PAHs (other parent compounds with significant toxicological and indicatory characteristics, 3.28 - 44.3 ng/m^3^) and oxidized PAHs (2.02 - 36.1 ng/m^3^) (Table 2). Assuming that traffic emitted n-alkanes and PAHs in similar proportions, then the approximately one order of magnitude higher PAH emissions in the hot spot site Bartovice is caused by emissions from other, mainly local industrial sources. Like n-alkanes, other contaminants associated with emissions from traffic (UCM, terpanes, triterpanes and steranes) were present in the samples. In addition to these compounds, sterols (mainly of plant origin, stigmasterol, *β*-sitosterol, β-amyrin, *β*-amyrin and lupeol), ubiquitous dialkyl-esthers of phthalic acid, which is still used as a softener for plastics based on polyvinyl chloride, and other industrial contaminants (bisphenol A, benzophenone, etc.) were also found. Different, more abundant individual U.S. EPA PAHs were found in the sites under study. Pyrene (a marker of pyrogenic sources of PAHs) dominated in Bartovice, Poruba and Trebon; Indeno[1,2,3-*cd*]pyrene (a marker of traffic sources) prevailed in the site Karvina (Table 3). The average concentration of benzo[*a*]pyrene ranged from 0.55 (Trebon site) to 5.98 ng/m^3 ^(hot spot site Bartovice). The difference in B[a]P was much higher in terms of B[a]P than in terms of PM2.5 (3-fold higher PM2.5 levels and more than 10-fold higher B[a]P levels in Ostrava-Bartovice and Trebon, respectively).

**Table 2:** Concentration of contaminant groups in the extracts from PM2.5 (ng/m^3^)

Contaminant classes	Contaminant groups	Ostrava-Bartovice	Ostrava-Poruba	Karvina	Trebon
**Polycyclic aromatic compounds**	U.S. EPA PAHs (10)*	76.3	13.6	17.1	5.89
	other PAHs (29)	44.3	7.14	9.29	3.28
	alkylated PAHs (46)	29.7	7.36	5.88	4.98
	oxidized PAHs (7)	36.1	11.0	12.8	2.02
	N-heterocyclic PAHs (PANHs) (13)	19.9	4.27	3.13	0.46
	S-heterocyclic PAHs (PASHs) (8)	9.99	2.27	1.80	0.42
	nitrated PAHs (15)	0.38	0.05	0.04	0.02
	dinitrated PAHs (3)	0.00097	0.00008	0.00005	0.00005
**Hydrocarbon markers**	n-alkanes (29)	84.4	89.5	77.4	89.2
	UCM (unresolved complex mixture) **	20.1	15.3	9.19	12.7
	terpanes (15)	14.8	9.34	5.64	4.14
	triterpanes (13)	4.49	3.16	1.49	3.73
	steranes (19)	2.24	2.68	0.85	1.76
**Sterols**	faecal sterols (8)	0.38	0.12	0.17	0.88
	phytosterols (5)	1.04	0.20	0.59	1.63
**Industrial contaminants**	musk compounds (9)	0.01	0.003	0.003	0.06
	dialkyl-phthalates (6)	0.75	0.60	0.34	2.08
	bisphenol A	0.18	0.09	0.10	0.09
	benzophenone	0.24	0.05	0.04	0.04
	isomyristate	0.19	0.10	0.07	0.46

* the numbers of quantified compounds are in parentheses

** mixture of thousands cyclic and branched saturated hydrocarbons forming a characteristic hump on the GC chromatogram of the fraction of non-polar compounds of air particulate matter

**Table 3:** Selected priority U.S. EPA PAHs adsorbed on the PM2.5 collected in various localities (ng/m^3^)

Compound name	Ostrava-Bartovice	Ostrava-Poruba	Karvina	Trebon
Fluoranthene	11.6	2.48	1.72	1.00
Pyrene	13.9	2.61	1.76	1.01
Benz[a]anthracene	11.6	1.62	1.67	0.50
Chrysene	9.06	1.96	2.19	0.89
Benzo[b]fluoranthene	3.89	0.55	1.03	0.31
Benzo[k]fluoranthene	4.34	0.69	1.18	0.32
Benzo[a]pyrene	5.98	1.31	2.26	0.55
Dibenz[a, h]anthracene	1.16	0.21	0.16	0.09
Benzo[ghi]perylene	5.15	0.80	1.83	0.51
Indeno[1,2,3-cd]pyrene	9.67	1.38	3.30	0.72

### Gene expression changes induced by EOMs

Gene expression profiling using the Illumina microarray platform and pathway analysis was used to identify deregulated genes and biological processes in HEL cells following 24 h exposure to EOM from each locality at three subtoxic concentrations (10, 30, 60 μg EOM/ml). Gene expression levels were compared to control HEL cell cultures treated with DMSO only.

### Deregulated transcripts and genes

**Figure 1:**
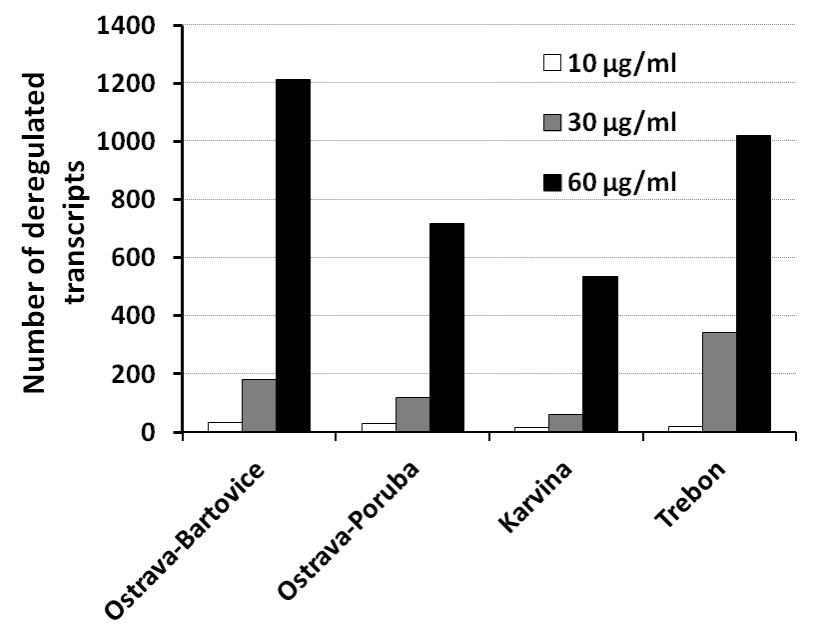
**Number of significantly deregulated transcripts (adjusted *P*-value < 0.05, average expression level (AvgExp) > 4, and log_2 _FC (fold change) > |1|) in HEL 12469 cells treated with various subtoxic concentrations of EOMs from PM2.5 collected in four localities of the Czech Republic**. List of all deregulated genes is available as Supplementary material.

**Figure 2:**
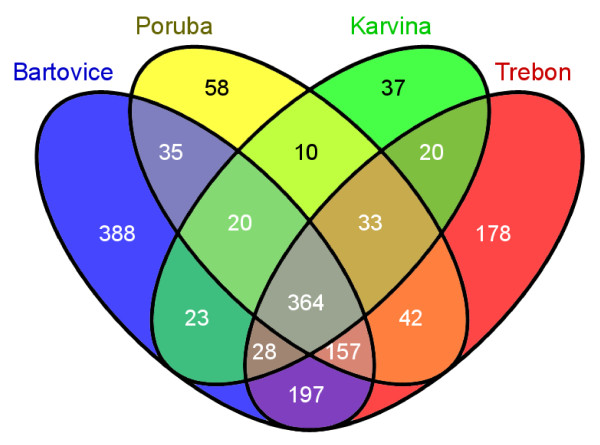
**Venn diagram representing numbers of common and locality-specific deregulated genes following 24-h treatment of HEL cells with 60 μg EOM/ml, relative to DMSO used as a solvent control (adjusted *P*-value < 0.05, average expression level (AvgExp) > 4, and log_2 _FC (fold change) > |1|)**.

We first identified differential gene expression in each EOM dose from all 4 localities. A full list of deregulated genes is available as Additional file 2. The number of deregulated transcripts with adjusted *P*-value < 0.05, average expression level (AvgExp) > 4, and log_2 _FC (fold change) > |1|exhibiting a positive dose response for all 4 localities is shown in Figure 1. More than 1200 transcripts were deregulated at the highest dose of 60 μg EOM/ml for the heavily polluted area of Ostrava-Bartovice, while after the exposure to the extract sample from Ostrava-Poruba (6 km from Ostrava-Bartovice) only about 700 genes were deregulated. Significant overlap of deregulated transcripts was observed between the localities (Figure 2). More than 360 transcripts were deregulated simultaneously in all 4 localities for EOM at the concentration of 60 μg EOM/ml. This number represented approximately 30% of all deregulated genes for Ostrava-Bartovice, 50% for Ostrava-Poruba, 68% for Karvina, and 36% for Trebon sample. Despite this significant overlap, 388 transcripts (32%) were exclusively deregulated in cells treated with EOM (60 μg/ml) from Ostrava-Bartovice, while only 58 (8%), 37 (7%), and 178 (18%) transcripts were deregulated by samples from Ostrava-Poruba, Karvina, and Trebon, respectively.

**Figure 3:**
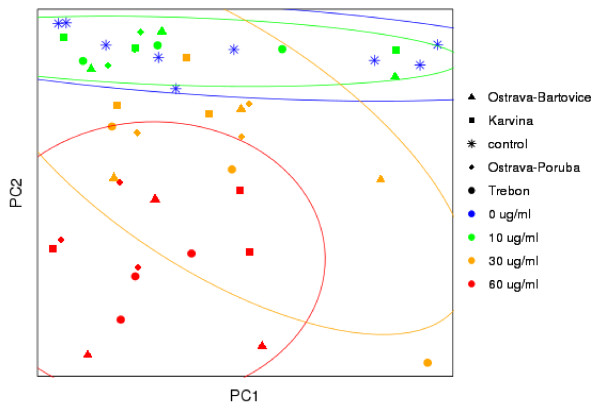
**Principal component analysis of the normalized gene expression data from microarrays for all localities and EOM concentrations. **Ellipses bound clusters of the various concentrations (95% confidence interval).

To further evaluate the differences in gene expression profiles between localities, principal component analysis (PCA) was performed (Figure 3). The data did not exhibit any significant clustering according to the locality, suggesting similarities in expression profiles. In contrast, clusters separating individual EOM concentrations were observed (ellipses in Figure 3 indicate 95% confidence interval). Further statistical analysis of the expression data was focused on the deregulated pathways involving the levels of all detectable transcripts, and not only significantly deregulated transcripts.

### Deregulated pathways

**Figure 4:**
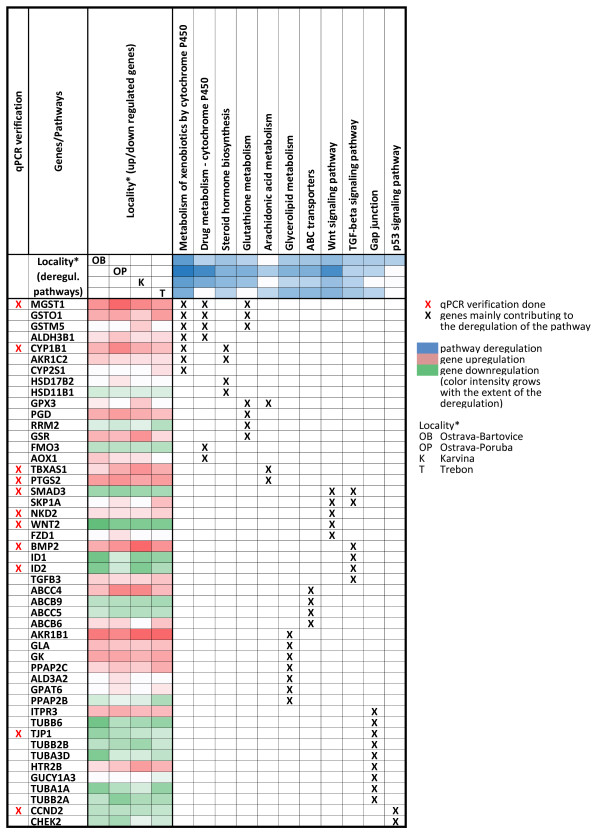
**Selected deregulated pathways and genes mainly contributing to their deregulation in various sampling localities**.

**Figure 5:**
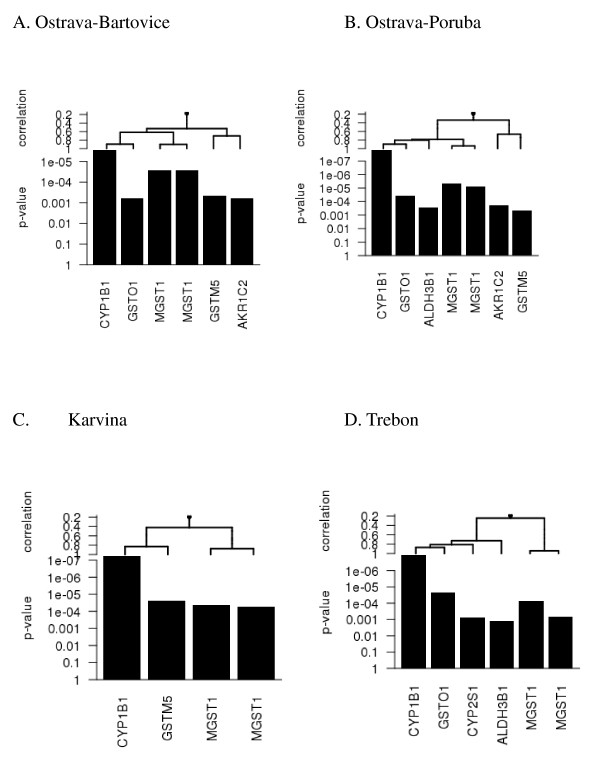
**Genes mainly contributing to deregulation of the KEGG pathway, metabolism of xenobiotics by cytochrome P450, in various sampling localities. **All depicted genes were upregulated.

To identify deregulated KEGG pathways for the individual localities, all 3 EOM concentrations (10, 30, and 60 μg/ml) were combined for the analysis. The main reason for that was to identify deregulated pathways in individual localities for the whole concentration range. The complete list of significantly deregulated pathways resulting from EOM-treated HEL 12469 cells, as identified by Goeman's global test, is shown in Table 4. Although the analysis on the level of individual transcripts identified an almost 2-fold higher number of deregulated genes for Ostrava-Bartovice than for Ostrava-Poruba (1212 vs. 719 for 60 μg EOM/ml), the number of deregulated pathways was higher for Ostrava-Poruba than for Ostrava-Bartovice (29 vs. 18). The pathway exhibiting the strongest deregulation in all 4 localities was the Metabolism of xenobiotics by cytochrome P450. The main genes contributing to the deregulation of this pathway included upregulation of *CYP1B1, MGST1 *(2 transcripts), *GSTM5*, and *GSTO1 *(Figure 4). The significance of this contribution as well as the correlations between transcripts are shown in Figure 5. These results suggest a crucial role of *CYP1B1 *upregulation and its correlation with the expression of other genes encoding detoxifying enzymes. All the genes depicted in Figure 4 were upregulated. Five other pathways were significantly deregulated in all 4 localities: Steroid hormone biosynthesis (driven mostly by *CYP1B1 *and aldo-ketoreductases), ABC transporters, Wnt signaling pathway, TGF-β signaling pathway, and Glycerolipid metabolism. The genes with the highest contribution to the deregulation of these pathways at various localities are summarized in Figure 4. Together with the pathways deregulated in all localities, Figure 4 summarizes 5 other toxicologically important pathways (Drug metabolism by cytochrome P450, Glutathione metabolism, Gap junction, Arachidonic acid metabolism, p53 signaling) deregulated in at least 1 of the 4 localities. For each pathway, selected genes mainly contributing to pathway deregulation are shown.

**Table 4:** Pathways significantly deregulated after EOM-treatment of HEL 12469 cells as identified by Goeman's global test

ID	KEGG pathway	Ostrava-Bartovice	Ostrava-Poruba	Karvina	Trebon
		Adj. p-value*			
**980**	**Metabolism of xenobiotics by cytochrome P450**	**4.06E-04**	**2.97E-05**	**4.71E-04**	**1.70E-03**
4270	Vascular smooth muscle contraction	**3.08E-04**	**2.06E-03**	*1.09E-01*	**2.41E-03**
**4310**	**Wnt signaling pathway**	**5.14E-03**	**1.32E-04**	**7.07E-03**	**2.88E-03**
30	Pentose phosphate pathway	**5.64E-03**	**8.84E-03**	*1.27E-01*	**3.19E-02**
**140**	**Steroid hormone biosynthesis**	**1.03E-02**	**2.58E-03**	**4.29E-03**	**3.37E-02**
**2010**	**ABC transporters**	**1.17E-02**	**1.86E-03**	**3.51E-02**	**4.34E-03**
**561**	**Glycerolipid metabolism**	**1.50E-02**	**2.63E-03**	**4.38E-02**	**5.03E-03**
770	Pantothenate and CoA biosynthesis	**1.64E-02**	**4.78E-02**	**3.68E-02**	*1.46E-01*
4540	Gap junction	**1.80E-02**	**3.50E-02**	*8.14E-02*	**3.63E-02**
**4350**	**TGF-beta signaling pathway**	**1.84E-02**	**3.07E-02**	**3.09E-02**	**1.29E-02**
4115	p53 signaling pathway	**2.39E-02**	*1.70E-01*	*2.74E-01*	7.77E-02
520	Amino sugar and nucleotide sugar metabolism	**2.58E-02**	**6.46E-03**	*1.22E-01*	*9.54E-02*
600	Sphingolipid metabolism	**2.63E-02**	*2.72E-01*	*8.92E-02*	**3.45E-02**
52	Galactose metabolism	**2.63E-02**	**1.97E-02**	*1.86E-01*	*7.77E-02*
620	Pyruvate metabolism	**2.89E-02**	**2.68E-02**	*1.00E+00*	**4.05E-02**
982	Drug metabolism - cytochrome P450	**3.01E-02**	**9.84E-05**	**1.49E-03**	*9.04E-02*
480	Glutathione metabolism	**3.08E-02**	2.13E-03	**7.91E-03**	*6.30E-02*
72	Synthesis and degradation of ketone bodies	**3.83E-02**	*2.10E-01*	*2.74E-01*	*1.00E+00*
4340	Hedgehog signaling pathway	*5.23E-02*	**2.22E-03**	**5.75E-02**	**4.33E-02**
5217	Basal cell carcinoma	*5.06E-02*	**5.04E-03**	**4.86E-02**	*5.16E-02*
40	Pentose and glucuronate interconversions	*6.10E-02*	**5.70E-03**	*1.59E-01*	*6.37E-02*
4142	Lysosome	*7.53E-02*	**5.78E-03**	*4.30E-01*	*7.05E-02*
565	Ether lipid metabolism	*5.47E-02*	**1.23E-02**	**1.52E-02**	**1.38E-02**
4614	Renin-angiotensin system	*5.43E-02*	**1.33E-02**	*1.22E-01*	*6.17E-01*
590	Arachidonic acid metabolism	*5.71E-02*	**1.97E-02**	*7.05E-02*	**4.70E-02**
4744	Phototransduction	*1.56E-01*	**2.07E-02**	*4.15E-01*	2.22E-02
511	Other glycan degradation	*1.09E-01*	**2.64E-02**	*5.96E-01*	*7.55E-02*
4610	Complement and coagulation cascades	*1.15E-01*	**2.85E-02**	*9.83E-01*	*4.56E-01*
5222	Small cell lung cancer	*1.48E-01*	**3.07E-02**	*4.63E-01*	*7.55E-02*
4612	Antigen processing and presentation	*4.26E-01*	**3.35E-02**	*5.52E-01*	*2.08E-01*
5140	Leishmaniasis	*1.18E-01*	**3.58E-02**	*2.20E-01*	*5.18E-02*
4145	Phagosome	*5.26E-02*	**4.38E-02**	*1.00E+00*	*1.16E-01*
564	Glycerophospholipid metabolism	*3.80E-01*	*1.08E-01*	*7.85E-01*	**6.10E-03**
4730	Long-term depression	*2.77E-01*	*4.64E-01*	*1.00E+00*	**7.49E-03**

KEGG pathways deregulated in all localities are in bold. PM2.5 samples were collected in 4 localities of the Czech Republic, and extractable organic matters (EOMs) were prepared as described in Materials and Methods.

*The procedure of Holm for control of the family-wise error rate [62].

### Quantitative real-time PCR verification

**Figure 6:**
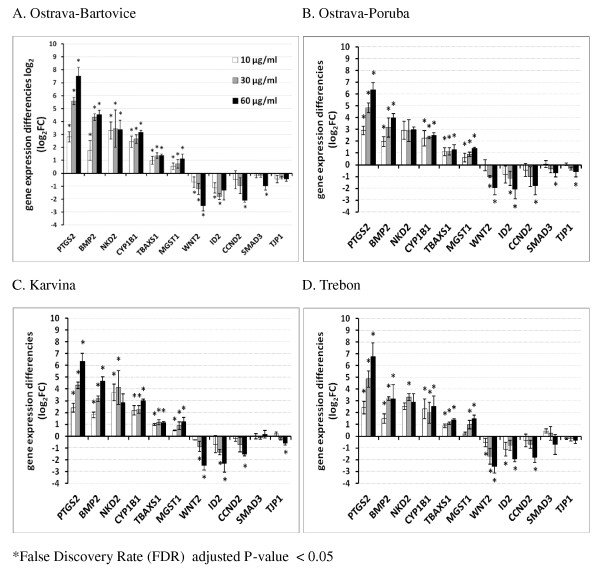
**Gene expression changes (gene expression difference log_2_) of 11 selected genes representing various deregulated pathways (see Table 4) as detected by quantitative RT-PCR**. HEL cells were treated with 10, 30 and 60 μg/ml of organic extracts from PM2.5 particles. All transcripts from all localities (with the exception of SMAD 3 for Karvina and Trebon and TJP1 for Trebon) were significantly deregulated in a dose-dependent manner (*P*-value of the Jonckheere-Terpstra monotonicity test was < 0.05).

**Figure 7:**
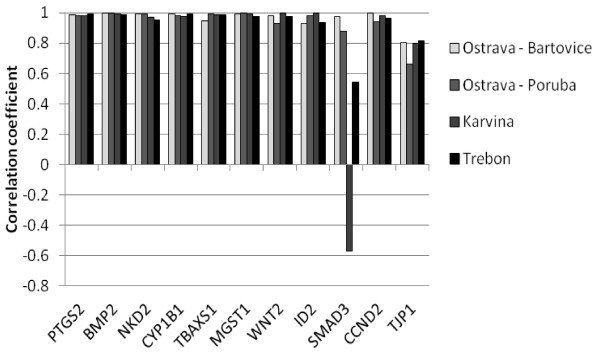
**Correlation of the expression of selected transcripts from microarrays and quantitative real-time PCR analysis**. The mean correlation across all the transcripts and localities was r = 0.91, (Pearson correlation coefficient).

The gene expression of 11 selected significantly deregulated genes from microarray data in HEL cells was verified by qPCR. These genes include *CYP1B1, MGST1, NKD2, BMP2, SMAD3, TBXAS1, CCND2, PTGS2, TJP1, WNT2*, and *ID2*. The transcripts were selected to represent various deregulated pathways (Figure 4). Transcript levels of each selected gene were measured in each locality (Figure 6 A-D). In most cases, data proved dose-dependent up- or downregulation as indicated by Jonckheere-Terprsta monotonicity test. With the exception of the downregulation of *SMAD3 *gene involved in *TGF-*β and Wnt signaling pathways, all other transcripts verified by qPCR were closely correlated (Figure 7). The mean correlation across all the transcripts and localities was r = 0.91, the mean correlation without *SMAD3 *gene was r = 0.96 (r is the Pearson correlation coefficient).

## Discussion

This study aimed to use human embryonic lung fibroblasts (HEL12469) as a model of target tissue for inhalation exposure, to identify biological processes and pathways involved in the toxic effects of organic extracts from respirable ambient air particles collected in 4 localities of the Czech Republic differing in the extent and sources of environmental pollution. For this purpose, cell cultures were treated with subtoxic concentrations of organic extracts from PM2.5 particles collected by high volume filter sampling. To ensure the complexity of the study, the whole genome RNA expression microarray covering 48 k of gene transcripts was used. The major findings of the study suggest that multiple genes involved in various biological pathways were deregulated in a dose-dependent manner, and the highest number of transcripts was deregulated in Ostrava-Bartovice, a residential part of Ostrava city which is mostly polluted by heavy industry, such as steel works and coke ovens located in the immediate vicinity [31]. Taking into account substantial differences in major air pollution sources among the localities, the high number of commonly deregulated genes seems to be surprising (30-68%, depending on the locality). This is further supported by the qualitative similarities in the chemical composition of the organic extracts from all 4 localities (determined more than 200 aromatic compounds), particularly by the results from the principal component analysis of gene expression profiles which indicated clustering according to the EOM concentration, but not according to the locality. However, this similarity was least for the most polluted area of Ostrava-Bartovice, where 32% of deregulated transcripts were exclusive to this locality, which was much more than for the 3 remaining areas. Chemical analysis revealed higher relative content of some of PAHs and nitrated PAH derivatives, which may at least explain a slightly different gene expression responses. However, the major modulations of gene expression were dependent on activation of aryl hydrocarbon receptor (AhR).

For better biological interpretation of the observed gene expression changes, we identified deregulated pathways without preliminary discrimination of up- and downregulated genes. The data indicated that some pathways were significantly affected by both up- and downregulated genes. We were also interested in pathways that contained a large number of genes whose regulation was associated with the EOM concentration in "a small way" (still under the significance threshold for each individual gene). Therefore, the preliminary discrimination of up- and downregulated genes may result in loss of some deregulated pathways. Furthermore, for the purpose of pathway analysis, we did not discriminate between individual EOM concentrations used in the treatment of HEL cells.

Taking into account the high levels of PAHs, their derivatives and many other compounds bound to PM2.5 [2], it is not too surprising that the strongest deregulation in this study was observed for the KEGG pathway, Metabolism of xenobiotics by cytochrome P450. Among the many upregulated metabolic enzymes in this pathway, *CYP1B1 *dominated in all localities. *CYP1B1 *is a mixed-function monooxygenase, which metabolizes mainly polycyclic aromatic hydrocarbons, N-heterocyclic amines, arylamines, aminoazodyes and several other carcinogens [32]. Besides its role in the metabolism of xenobiotics, *CYP1B1 *is also involved in the metabolism of cholesterol, steroid hormones, arachidonic acid and other lipids, metabolism of retinoic acid as well as in vitamin D3 synthesis and metabolism [33].

The induction of *CYP1B1 *by complex mixtures such as tobacco smoke or airborne particles has been observed [14,25,34]. It is well known that the *CYP1B1 *gene is under the regulatory control of the AhR and many PAHs are known to induce *CYP1B1 *and their own metabolism through binding to and activation of the AhR [35]. The AhR is a ligand-activated transcription factor which has a central role in the induction of drug-metabolizing enzymes. AhR can also interact with other pathways suggesting that this activity is important in the toxicity of exogenous compounds. AhR activation by some of its ligands participates, among others, in pathways involved in the oxidative stress response, cell cycle control and apoptosis [36], cell adhesion and matrix remodeling [37] as well as multiple developmental pathways [19]. Strong involvement of AhR related pathways in the toxic response of EOMs within this study are supported by recent findings on the mechanisms of toxicity induced by an organic extract of the urban dust standard reference material, SRM1649a [14], also suggesting a crucial role for AhR and PAHs as key AhR activators. Another group of upregulated genes within the metabolism of xenobiotics by cytochrome P450 pathway, are glutathione S-transferases *MGST1 *and *GSTM5*, known to be involved in conjugation of reduced glutathione to a wide number hydrophobic electrophile metabolites [38,39], and *GSTO1*, a glutathione-dependent thiol transferase and dehydroascorbate reductase [40].

In this study, *CYP1B1 *upregulation was also a major factor in deregulation of the second most important deregulated KEGG pathway - Steroid hormone biosynthesis (SHB), which is known to be a target for endocrine-disrupting chemicals [41]. Similar to the Metabolism of xenobiotics by cytochrome P450, this pathway was deregulated in all 4 localities. In addition to *CYP1B1*, which is known to hydroxylate estrogens [42], aldo-keto reductase (*AKR1C2*) and some hydroxysteroid β-dehydrogenases 1 (*HSD17B2, HSD11B1, HSD17B8*) significantly contributed to deregulation of the SHB pathway. The results of the detailed chemical analysis of the EOMs, including the analysis of the dioxin toxicity of the fractionated crude extracts, strongly suggests that PAHs (abundant components in all EOMs) and not persistent organic pollutants (chlorinated dibenzo-p-dioxins, dibenzofuranes or biphenyls) are mainly responsible for SHB deregulation and dioxin-like toxicity. These findings are in accordance with our previous study [14], in which the activation of AhR and AhR-mediated gene expression and cellular nongenotoxic events prevailed the genotoxic and apoptotic processes. Several mechanisms on the modulation of estrogen and androgen signaling by chemicals directly or indirectly through cross-talk between AhR and steroid hormone receptors have been discussed [43,44]. Here we found a possible correlation between the modulations of enzymes of steroidogenesis and oxidative steroid metabolism and the AhR activation.

The crucial role of AhR in the toxic effects of the EOM components is further underlined by the analysis of the third KEGG pathway - Wnt signaling, which was significantly deregulated by all 4 extracts in this study. Wnt signaling proteins are required for basic developmental processes in many different organs. A recent genomic analysis revealed functional cross-talk between AhR and the well-established Wnt/β-catenin signal transduction pathway [45,46]. *NKD2*, as an upregulated gene mostly contributing to the deregulation of Wnt signaling in all EOM-treated cells, is known as a cell autonomous antagonist of the canonical Wnt signaling pathway [47]. Accordingly, Wnt target genes, *WNT2 *and *CCND2 *were significantly downregulated in lung fibroblasts exposed to all 4 extracts (Figure 6).

The next KEGG pathway deregulated by EOMs from all 4 localities was the Transforming growth factor-β(*TGF-β*) signaling, which includes structurally related cytokines regulating a wide spectrum of cellular functions such as cell growth and proliferation, apoptosis, differentiation and migration via receptors type I and II [48]. In our study, the upregulation of bone morphogenic protein type-2 (*BMP2*), antagonist of TGF-β involved in osteogenesis, cell differentiation, growth and invasivity, is primarily responsible for TGF-β signaling deregulation [49]. Simultaneously, downregulation of *SMAD3*, effector of TGF-β signaling, and DNA-binding inhibitors 1 or 2 (*ID1, ID2*), transcription factors which negatively regulate cell differentiation [50], was observed in this study. Again, suppression of TGF-β signaling by activated AhR has been reported [51], pointing out the key role of AhR activation in lung fibroblasts exposed to complex airborne mixtures.

The ATP binding cassette (ABC transporters), as a fifth pathway deregulated by all extracts in this study, includes a huge number of various transmembrane proteins capable of active transport of various compounds through the cell membrane. In humans, there are 49 known ABC transporters, which are classified into eight families [52]. In our study, the most significant deregulation was observed for family C (C4 and C5), known to facilitate transport of bile salt and steroid conjugates, ion transport and toxin excretion activity, and family B (B6 and B9), used mostly for transport of peptides [53]. Recently, AhR-dependent upregulation of *ABCC4 *was reported [54]. In eukaryotes including humans, ABC transporters serve as pumps that extrude toxins from the cell. Some ABC proteins are known to be involved in translation and DNA repair processes. It was obvious that exposure of HEL cells to complex mixtures of organic compounds bound to PM2.5 induced deregulation of many ABC transporters as a defending reaction of cells to this exposure and that AhR induction may play a significant role in their upregulation. Similar primary transcription response was reported in mouse lung fibroblasts exposed to TCDD for 4 h [55]. TCDD-induced significant upregulation of *CYP1B1, PTGS2, BMP2, ABCC4 *and deregulation of other genes belonging to Metabolism of xenobiotics, ABC transporters, TGF-beta and Wnt signaling pathways in mouse lung fibroblasts suggests a significant AhR-dependent gene expression in the HEL cells.

The last pathway deregulated in all 4 localities was Glycerolipid metabolism, which includes the chemical reactions and pathways involving glycerolipids, the lipid with a glycerol backbone. Diacylglycerol and phosphatidic acid are key lipid intermediates of glycerolipid biosynthesis; diacylglycerol is a key lipid signaling molecule involved in activation of protein kinases and cell survival and proliferation. The deregulation of this pathway is caused mainly by upregulation of aldo-keto reductase 1B1 (*AKR1B1*) catalyzing the NADPH-dependent reduction of a wide variety of carbonyl-containing compounds to their corresponding alcohols with a broad range of catalytic efficiencies [56]. It is very likely that carbonyl compounds are the components of all 4 EOMs. Importantly, genes responsible for sphingolipid metabolism were also significantly deregulated; these results suggest effects on sphingolipid signaling molecules which regulate cell survival, proliferation and apoptosis.

There were many other deregulated pathways detected in this study (Table 5), but these were not found in all localities, e.g. Glutathione metabolism pathway was deregulated after the treatment with the extracts from sampling sites Ostrava-Bartovice, Ostrava-Poruba and Karvina but not after the exposure to extract from Trebon, the only agricultural area. The genes in the KEGG cluster of Glutathione metabolism belong to the phase II biotransformation of xenobiotics and oxidative stress defense. The genes of arachidonic metabolism are involved in proinflammatory responses (*PTGS2, TBXAS1*) and protection against oxidative stress (*GPX3*). Interestingly, the p53 signaling pathway was deregulated exclusively after the treatment by EOM from Ostrava-Bartovice, the most polluted industrial locality. It was found no induction of p53 target genes suggesting possible suppresive role of activated AhR [20]. Genes mostly involved in the deregulation of the Gap junction pathway belong rather to microtubule functions, mitosis and tight junction, they are not so relevant for gap junctions. In conclusion, major deregulated KEGG pathways are related to various cancer promoting processes.

**Table 5:** Sequences of primers used in quantitative RT-PCR

Symbol	RefSeq ID	Oligonucleotide	
CYP1B1	NM_000104.2	sense	CACTGGAAACCGCACCTC
		antisense	AGCACCGACAGGAGTAGC
MGST1	NM_145792.1	sense	CACCTGAATGACCTTGAAAATATTATT
		antisense	TCCGTGCTCCGACAAATAGT
NKD2	NM_033120.2	sense	GGAAGGTCACCAGGGAGGA
		antisense	TTCACACGGAGGGTCTTGC
BMP2	NM_001200.2	sense	GGGCATCCTCTCCACAAAAG
		antisense	CCACGTCACTGAAGTCCAC
SMAD3	NM_005902.3	sense	GGCTGCTCTCCAATGTCAAC
		antisense	ACCTCCCCTCCGATGTAGTA
TBXAS1	NM_001061.2	sense	ATCTTCCTCATCGCTGGCTAT
		antisense	CCTTAAAAACGTCTACCTCTCCA
CCND2	NM_001759.2	sense	TGGGACAATGGGTGGTGAA
		antisense	GCAAAGCTGGCTCTTGAGAA
PTGS2	NM_000963.1	sense	CAAATCATCAACACTGCCTCAAT
		antisense	TCTGGATCTGGAACACTGAATG
TJP1	NM_175610.2	sense	AAACAAGCCAGCAGAGACC
		antisense	CGCAGACGATGTTCATAGTTTC
WNT2	NM_003391.1	sense	CAAGAACGCTGACTGGACAA
		antisense	CCCCAGAAAGAACCCAAAGG
ID2	NM_002166.4	sense	CGATGAGCCTGCTATACAACA
		antisense	AGGTCCAAGATGTAGTCGATGA

### Limitations of this study

The major limitation of this study was that the comparison of the various localities, in terms of the gene expression profiles, should only be regarded as qualitative since equal EOM doses used for all localities (10-60 μg EOM/ml) did not reflect different EOM content per m^3 ^of the sampled air. In contrast to some toxicity markers such as stable DNA adduct formation [1], gene expression data cannot be normalized to EOM/m^3^. Therefore, to make a quantitative comparison of the effect of organic compounds bound to PM2.5 on gene expression profiles, the EOM doses used for cell treatment should take into account the differences in EOM/m^3^. Such a study is in progress. On the other hand, using of equal EOM doses allowed us to reveal similar gene expression profiles and affected KEGG pathways.

For technical reasons, it was impossible to sample PM2.5 simultaneously in all 4 localities, which is another limitation of the study. This fact may partially explain why the agricultural locality of Trebon sampled in November and December (period of frequent winter inversions) exhibited such a high number of deregulated transcripts compared to Ostrava-Poruba and Karvina, industrial locations sampled in March and April, respectively. The effect of the winter inversions on particulate matter and PAH air pollution is well known [57].

## Conclusion

To our knowledge, this is the first study dealing with differential gene expression in the context of real complex mixtures of air pollutants at the level of the whole genome in human lung fibroblasts. The study identified KEGG pathways deregulated by real complex mixtures of air pollutants collected in areas differing in the extent and sources of air pollution and the key role of activation of AhR was found. The results of this study may be used for future more detailed mechanistic studies focused on the role of individual affected pathways and genes.

## Materials and methods

### Reagents

All chemical standards were purchased from Promochem (Wesel, Germany), Dr. Ehrenstorfer (Augsburg, Germany) or Midwest Research Institute (Kansas City, MO, USA); solvents were from Merck (Darmstadt, Germany) and chromatographic consumables from Sigma-Aldrich (Prague, Czech Republic). The other compounds and materials used were of the highest purity available suitable for organic trace analysis. DMSO was purchased from Merck, Darmstadt, Germany. The sources of other specific chemicals and kits are indicated below.

### PM2.5 collection, sampling sites and EOM extraction

Particulate matter < 2.5 μm (PM2.5) was collected by a HiVol 3000 air sampler (model ECO-HVS3000, Ecotech, Australia) on Pallflex filters T60A20 (20 × 25 cm) in four localities of the Czech Republic differing in the extent and major sources of air pollution: Ostrava-Bartovice (heavily polluted industrial area), Ostrava-Poruba (high level of traffic), Karvina (industrial area) and Trebon (rural area with some houses equipped with local brown coal heating) as described by Topinka et al. [31]. Briefly, sampling was conducted for 24 h each day for 30-35 days in the winter season of 2008/2009. Each filter was extracted by 60 ml of dichloromethane and 3 ml of cyclohexane for 3 hours. The extracts (EOMs) from all filters with PM2.5 samples were pooled and aliquots were used for the detailed chemical analysis and the cell treatment. The extraction of PM2.5 was performed in the laboratories of the certified company ALS Czech Republic, Prague (EN ISO CSN IEC 17025). For the *in vitro *experiments, EOM samples were evaporated to dryness under a stream of nitrogen and the residue re-dissolved in dimethylsulfoxide (DMSO). The stock solution of each EOM sample contained 50 mg of EOM/ml DMSO. Samples were kept in the freezer at -80°C until analysis.

### Sample handling for chemical analysis

Extracts of air PM_2.5 _samples were used for fractionation into four fractions using low-pressure silica gel column chromatography. Fractionation was performed to facilitate the chemical analysis of complex mixtures of polar and nonpolar contaminants of the air samples. An aliquot of the sample extract in dichloromethane was evaporated just to dryness; the residues was redissolved in 0.5 ml of hexane and applied to the top of the open silica gel column. The silica gel (Silica gel 60, particle size 0.063-0.2 mm, Merck, Darmstadt, Germany) was activated for 1 hour at 200°C prior to its use. A column with the dimensions 250 × 10 mm was dry-packed with 10 g of activated silica gel and washed with 30 ml of hexane prior to the application of the sample. Fractionation was done by gradual elution with 20 ml of hexane to obtain an aliphatic fraction (this fraction was used for alkanes, terpanes and steranes analysis), followed by 20 ml of hexane/dichloromethane (1:1, v/v) (fraction including parent aromatic and POPs compounds), 20 ml of dichloromethane (fraction with slightly-polar compounds such as nitrated derivatives of PAHs) and finally by 30 ml of methanol (polar compounds represented by oxygenated derivatives of PAHs, heterocyclic PAHs with one atom of nitrogen, esters of phthalic acid and sterols). Aliquots of these fractions were redissolved in the required volume of acetonitrile for HPLC/DAD, LC/MS-MS and in 2,2,4-trimethylpentane for GC/MS analysis.

### HPLC, LC/MS-MS and GC/MS analysis

The HPLC system consisted of a Waters 717 plus autosampler, a Waters 600 E multisolvent delivery system, a Waters 474 scanning fluorescence detector and a Waters 996 photodiode array detector (Waters, Milford, MA, USA). A 150 × 3 mm Supelcosil LC-PAH column with particle diameter 5 μm (Supelco, Bellefonte, PA, USA) was used for the separation of parent PAHs with molecular weights (MW) ranging from 178 to 326 g/mol. A gradient with water, methanol, acetonitrile and tetrahydrofuran was applied to separate the analytes: 0-55 min. 40-0% water, 30% acetonitrile and 30-70% methanol, 55-72 min. 30-100% acetonitrile and 70-0% methanol, 72-100 min. 100-72% acetonitrile and 0-28% tetrahydrofuran. The flow rate of the mobile phase was 0.6 ml/min., the column temperature was set at 35°C.

The LC/MS-MS analysis of parent PAHs (178-326 MW) and nitrated and oxygenated derivatives of PAHs was performed on a TripleQuad 6410 triple quadrupole mass spectrometer (Agilent, Santa Clara, CA, USA) equipped with an electrospray ion source (ESI), an Agilent 1200 Binary Pump System with an autosampler and a MassHunter software system. The ionization of the analytes was performed in the positive ion mode. The analyte classes were separated in a reverse-phase mode using a Supelcosil LC-PAH HPLC column (150 mm × 3 mm, 5 μm - Supelco, Bellefonte, PA, USA).

Other classes of contaminants included hydrocarbon markers, parent PAHs (128-278 MW), alkylated, oxidized and nitrated derivatives of PAHs and compounds with one heterocyclic atom in the ring (PANHs, PASHs) were determined by GC/MS. GC separation was done in a fused silica capillary column (SLB-5 ms: 30 m × 0.20 mm × 0.20 μm - Sigma-Aldrich, Prague, Czech Republic) with helium as the carrier gas. A Saturn 2100 T ion trap mass spectrometer (Varian, Walnut Creek, CA, USA), which operated in electron ionization and selected ion storage modes at an electron ionization energy of 70 eV, was used for the identification and quantification of the analytes under study.

### Cell cultures and cytotoxicity

Human embryonic lung diploid fibroblasts (HEL 12469a, ECACC, UK) were grown in minimal essential medium E-MEM supplemented with 10% FBS, 2 mM glutamine, 1% non-essential amino acids, 0.2% sodium bicarbonate, 50 U/ml penicillin and 50 μg/ml streptomycin. The cells were cultivated in plastic cell culture dishes (21 cm^2^) at 37°C in 5% CO_2_. After reaching 90% confluency, the medium was replaced with fresh medium supplemented with 1% FBS. EOM samples were diluted by DMSO and added to the medium at the test concentrations: 10, 30 and 60 μg/ml. The cells were treated for 24 h. Each concentration was tested in triplicate including control cell cultures incubated with DMSO only. The harvested cells were washed three times in PBS and the final concentration of DMSO did not exceed 0.1% of the total incubation volume. The cytotoxicity of the extracts in HEL cells was tested by the LDH-Cytotoxicity Assay Kit (Bio Vision, catalogue #K311-400) at concentrations 10, 30, 60 and 100 μg EOM/ml. Significant cytotoxicity was observed at the highest EOM concentration of 100 μg/ml for extracts from Ostrava-Bartovice and Trebon (66% and 17%, respectively). Therefore, three subtoxic EOM concentrations between 10 and 60 μg/ml were used.

### RNA isolation and quality control

Total RNA from lysed HEL cells was obtained using NucleoSpin RNA II (Macherey-Nagel GmbH & Co.KG, Düren, Germany) according to the manufacturer's instructions. RNA concentration was quantified with a Nanodrop ND-1000 Spectrophotometer (Thermo Fisher Scientific, Waltham, MA, USA). The integrity of RNA was assessed using an Agilent 2100 Bioanalyzer (Agilent Technologies Inc., Santa Clara, CA, USA). All samples had an RNA Integrity Number (RIN) above 9. Isolated RNA was stored at -80°C until processing.

### Gene expression profiling and data analysis

Illumina Human-HT12 v3 Expression BeadChips (Illumina, San Diego, CA, USA) were used to generate expression profiles. Biotinylated cRNAs were prepared from 200 ng of total RNA using the Illumina TotalPrep RNA Amplification Kit (Ambion, Austin, TX, USA). Next, 750 ng of biotinylated cRNA targets was hybridized to the beadchips. The steps of hybridization and the subsequent washing, staining and drying of the beadchips were processed according to standard instructions from Illumina. The hybridized beadchips were then scanned on the Illumina BeadArray Reader and bead level data were summarized by Illumina BeadStudio Software v2.

### Quantitative RT-PCR verification

Two thousands ng RNA from each sample was used for cDNA synthesis using the High Fidelity cDNA synthesis Kit (Roche, Manheim, Germany). The original protocol was modified by using 2.5 μM oligo(dT) and 10 μM random hexamers for priming in a 20 μl reaction volume. cDNA synthesis was run according to the following conditions: 30 min at 55°C and 5 min at 85°C. Quantitative PCR measurements were performed using the 7900 HT Fast Real-Time PCR System (Applied Biosystems, Carlsbad, CA, USA). Each qPCR reaction was carried out in a final volume of 14 μl containing 3.5 μl of diluted cDNA, 2.8 μl of water and 7 μl of master mix (Primerdesign, Southampton, UK). To determine the level of each target gene, 0.7 μl of a specifically designed assay (PerfectProbe, Primerdesign) was added to the reaction mixture (list of primers in Table 5). Cycling conditions were: 10 min at 95°C followed by 40 cycles of amplification (15 s at 95°C, 30 s at 50°C and 15 s at 72°C). Raw data were analyzed with SDS Relative Quantification Software version 2.3 (Applied Biosystems, USA) to assign the baseline and threshold for Ct determination. The sequences of primers used in quantitative RT-PCR are shown in Table 5.

### Statistical analysis

Gene expression levels were compared with control HEL cell cultures treated with DMSO only. Bead summary data were imported into R statistical environment http://www.r-project.org and normalized using the quantile method in the Lumi package [58]. Only probes with a detection *P*-value < 0.01 in more than 50% of arrays were included for further analyses. Differential gene expression was analyzed in the Limma package using the moderated *t*-statistic. A linear model was fitted for each gene given a series of arrays using lmFit function [59]. Multiple testing correction was performed using the Benjamini & Hochberg method. A Venn diagram was prepared according to Oliveros [60].

Goeman's global test [61] and the KEGG database were applied to identify deregulated biological pathways and deregulated genes within these pathways. The procedure of Holm for control of the family-wise error rate was applied [62]. The Jonckheere - Terpstra monotonicity test [63,64] was used to analyze the dose response of expression of selected genes.

Ct values of real-time PCR data were analyzed using GenEx software version 5.2.7 (MultiD Analyses AB, Goteborg, Sweden). The expression levels of the target genes were normalized to the expression levels of the reference genes *GAPDH *and *SDHA*. Reference genes were selected according to the stability of gene expression during experimental conditions using the geNorm reference gene selection kit (Primerdesign).

## List of abbreviations

ABC: ATP binding cassettes; AhR: aryl hydrocarbon receptor; B[a]P: benzo[a]pyrene; DCM: dichloromethane; EOM: extractable organic matter; HPLC: high performance liquid chromatography; PAHs: polycyclic aromatic hydrocarbons; PM2.5: particulate matter < 2.5 μm; KEGG: Kyoto Encyclopedia of Genes and Genomes; LDH: lactate dehydrogenase; qPCR: quantitative real time PCR; RIN: RNA integrity number, TGF-β: transforming growth factor beta; SHB: steroid hormone biosynthesis.

## Competing interests

The authors declare that they have no competing interests.

## Authors' contributions

HL carried out gene expression analysis on Illumina microarrays and qPCR verification. She also substantially contributed to the description of results and their discussion and interpretation. KU carried out cell preparations and treatment, cytotoxicity analysis and gene expression on Illumina microarrays. JK was responsible for biostatistical evaluation of data. MM substantially contributed to the data interpretation. RJS performed overall text revision, and he contributed to the Discussion. MC was responsible for detailed chemical analysis of extracts from particulate matter. JT was responsible for PM2.5 sampling, EOM extraction, chemical analysis and the overall preparation of the manuscript. All authors read and approved the final manuscript.

## Supplementary Material

Additional file 1 **Supplementary table with the list of chemical compounds identified and quantified in EOMs from various localities**. Click here for file

Additional file 2 **Complete list of significantly deregulated genes in HEL cells treated with 10, 30, and 60 **μ**g/ml of organic extracts from PM2.5 collected in Ostrava-Bartovice, Ostrava-Poruba, Karvina, and Trebon**. Each excel sheet contains list of deregulated transcripts detected from the comparison of gene expression profile of cells treated with an appropriate EOM (Table 1) and cells treated with DMSO. Transcripts with adjusted p-value > 0.05 and average expression < 4 were filtred out. Click here for file
